# Temporal changes in treatment patterns by age group and functional status before and after PD-1/L1 inhibitor approvals in advanced urothelial carcinoma

**DOI:** 10.3389/fonc.2023.1210208

**Published:** 2023-10-02

**Authors:** Gurjyot K. Doshi, Haojie Li, Mehmet Burcu, Srinivas Annavarapu, Karen Wells, Kentaro Imai, Blanca Homet Moreno, Puneet Singhal, Ronac Mamtani

**Affiliations:** ^1^ The US Oncology Network, The Woodlands, TX, United States; ^2^ Merck & Co., Inc., Rahway, NJ, United States; ^3^ Ontada, The Woodlands, TX, United States; ^4^ IQVIA, New York, NY, United States; ^5^ Division of Hematology and Oncology, Department of Medicine, University of Pennsylvania, Philadelphia, PA, United States

**Keywords:** treatment pattern, advanced urothelial carcinoma, real-world, observational study, US, immunotherapy

## Abstract

**Introduction:**

Metastatic urothelial carcinoma (mUC) has poor prognosis. A high unmet need exists for novel treatment for those who are unfit for platinum-based chemotherapy.

**Methods:**

We aimed to describe real-world temporal changes in patient characteristics and 1L treatment selection for mUC patients in the United States following the approval of anti-PD-1/L1 treatments. This study was a retrospective, observational study using anonymized and structured oncology electronic medical record (EMR) data from IQVIA and the US Oncology Network iKnowMed (USON).

**Results:**

After approval of 1L anti-PD-1/L1 treatment for mUC, there is a marked increase in the use of 1L anti-PD-1/L1 monotherapies, accompanied by a proportional decrease in 1L platinum-based treatments and non-guideline-based therapy; particularly among the elderly (> 75 years) and those with poor ECOG performance status (ECOG PS 2+).

**Discussion:**

Anti-PD-1/L1 monotherapies fulfill the prior unmet need of frail mUC patients who are ineligible for platinum-based therapies.

## 1 Introduction

Metastatic urothelial carcinoma (mUC) disproportionately affects the elderly and typically is incurable, with poor prognosis. Platinum-containing chemotherapy remains the first-line (1L) standard-of-care treatment for mUC patients (1).

Approximately 40-60% of 1L mUC patients are not eligible for cisplatin-based chemotherapy due to poor performance status (ECOG PS) or renal impairment (2–4). Many 1L mUC patients are not eligible for carboplatin-based chemotherapy, due to age-associated comorbidity or medical frailty, or decline treatment due to perceptions of treatment-related toxicity (5). For platinum-ineligible mUC patients, no approved drug was available until the approval of anti-PD-1/L1 immuno-oncology (IO) treatments. As such, many 1L mUC patients were not offered any treatment or were treated with non-guideline-based therapies (2–4).

Pembrolizumab and atezolizumab were approved in 2017 and are currently recommended by The NCCN Clinical Practice Guidelines in Oncology (NCCN Guidelines®) for Bladder Cancer (v3.2023) (6) as monotherapies specifically in the setting of 1L cisplatin-ineligible (atezolizumab only for patients whose tumors express PD-L1) or platinum-ineligible patients (pembrolizumab and atezolizumab irrespective of PD-L1 status). Very limited prior data have described the impact of IO treatment availability on 1L treatment landscape among mUC patients. This study aimed to describe real-world temporal changes in patient characteristics and 1L treatment selection following the approval of anti-PD-1/L1 treatments for mUC.

## 2 Methods

This study was a retrospective, observational study using anonymized and structured oncology electronic medical record (EMR) data from IQVIA and the US Oncology Network iKnowMed (USON). IQVIA Oncology EMR contains data from multiple oncology specific EMR data sources across a consortium of health care provider locations in the US that include community practices, hospitals, and academic medical centers (>1.1 million oncology patients across 41 states). The US Oncology Network iKnowMed (USON) EMR is an oncology-specific system that captures outpatient community practice encounter history for patients under care within the US Oncology Network (~10% of newly diagnosed cancer patients in the US annually across 19 states and 480 sites).

The study included patients (≥18 years of age) with a diagnosis of mUC (during 01/01/2012-09/30/2020 in IQVIA EMR & 01/01/2010-09/30/2020 in USON) who had ≥2 visits and initiated 1L systemic anticancer therapy. Patients were excluded if they had a non-UC primary tumor (excepting non-melanoma skin cancer and *in-situ* or benign neoplasms). First-line systemic treatments were grouped as: cisplatin-based chemotherapy, carboplatin-based chemotherapy, anti-PD-1/L1 monotherapy (pembrolizumab, atezolizumab, and others), and other treatments (including non-carboplatin/non-cisplatin platinum-based chemotherapy, non-platinum chemotherapy, anti-PD-1/L1combination with chemotherapy, and non-chemotherapy/non-immunotherapy treatment). The first regulatory approval of an anti-PD-1/L1 treatment for 1L advanced/metastatic UC occurred in April 2017. The analyses were conducted separately in two distinct time-periods (before and in/after April 2017) and included descriptive statistics of demographic and clinical characteristics, and 1L treatment choice, overall and by age and ECOG performance score at 1L treatment start.

## 3 Results

**Table S1** describes the demographics and clinical characteristics of 1L-treated patients with mUC from two Oncology EMR databases before (pre-IO) and on/after (post-IO) regulatory approvals of anti-PD-1/L1 treatments in the 1L setting. Compared to the pre-IO period, there was an increase in the proportion of older adults (age 75+: USON, 41.3% vs. 37.5%; IQVIA, 45.0% vs. 37.0%) and with poor ECOG PS (2+: USON, 24.6% vs. 19.1%; IQVIA, 20.7% vs. 14.5%) in the post-IO period.

**Table 1** describes 1L treatment patterns in the pre-IO and post-IO time-periods separately in IQVIA and USON EMR databases. Among mUC patients who initiated 1L treatment, compared with pre-IO period, the proportion of patients with platinum-based treatments decreased sharply in the post-IO period. For example, in the post-IO period (compared with pre-IO period), the proportion of cisplatin-based regimens decreased from 36.5% to 22.7% in USON and from 42.5% to 31.3% in IQVIA. Similar proportional decreases were also observed for carboplatin-based regimens in post-IO period compared with pre-IO period (USON, from 35.5% to 17.5%; IQVIA, from 29.4% to 22.2%). By contrast, there was a proportional marked increase in the use of anti-PD-1/L1 monotherapy in the post-IO period compared with pre-IO period (USON, from 3.7% to 52.8%; IQVIA, from 14.4% to 37.4%). The most utilized 1L anti-PD-1/L1 treatment was pembrolizumab (USON, 30.0%; IQVIA, 21.2%) followed by atezolizumab (USON, 18.4%; IQVIA, 11.8%) in the post-IO period. Use of non-NCCN guideline recommended (other) became much less common in the post-IO vs. pre-IO period (USON, from 24.3% to 7.0%; IQVIA, from 13.7% to 9.1%).

**Table 1:** Treatment pattern among 1L treated advanced urothelial carcinoma patients.

	US Oncology (USON) EMR		IQVIA Oncology EMR	
	(Jan 2010-Mar 2017)	(Apr 2017-Sep 2020)	(Jan 2012-Mar 2017)	(Apr 2017-Sep 2020)
All 1L Treated patients	1508 (100%)	928 (100.0%)	1130 (100.0%)	1391 (100.0%)
Cisplatin-based regimen	551 (36.5%)	211 (22.7%)	480 (42.5%)	436 (31.3%)
Carboplatin-based regimen	534 (35.5%)	162 (17.5%)	332 (29.4%)	309 (22.2%)
IO monotherapy	56 (3.7%)	490 (52.8%)	163 (14.4%)	520 (37.4%)
Pembrolizumab	4 (0.3%)	278 (30.0%)	31 (2.7%)	295 (21.2%)
Atezolizumab	49 (3.2%)	171 (18.4%)	71 (6.3%)	164 (11.8%)
Other IO	3 (0.2%)	41 (4.6%)	61 (5.4%)	61 (4.4%)
Other^1^	367 (24.3%)	65 (7.0%)	155 (13.7%)	126 (9.1%)

Other^1^ included other platinum chemo, non-platinum chemo, IO-chemo combo, and non-chemo/non-IO treatment.

**Figure 1:**
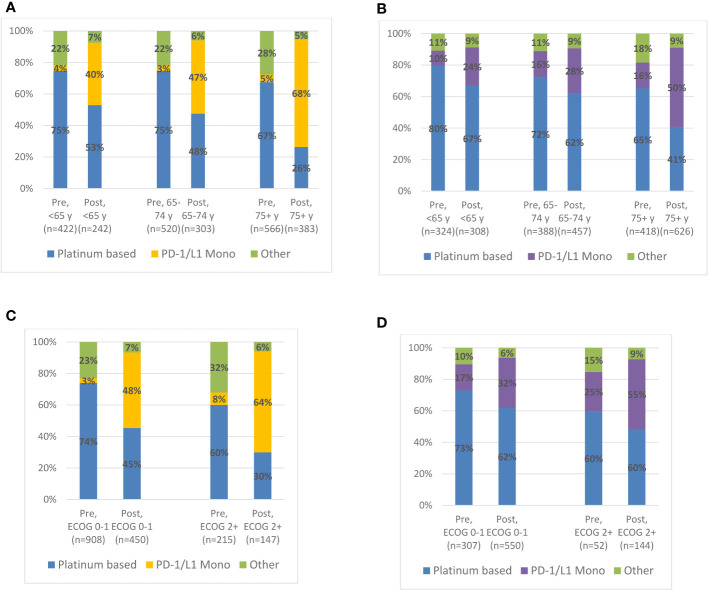
Treatment Patterns by Age or ECOG PS Status at 1L Treatment Initiation among Advanced Urothelial Carcinoma Patients in the Pre-IO (US Oncology EMR, Jan 2010-Mar 2017; IQVIA Oncology, Jan 2012-Mar 2017) and Post-IO (Apr 2017-Sep 2020) Eras. **(A)** US Oncology (USON) EMR: 1L treatment patterns by age category. **(B)** IQVIA Oncology EMR: 1L treatment patterns by age category. **(C)** US Oncology EMR: 1L treatment patterns by EOCG PS status. **(D)** IQVIA Oncology EMR: 1L treatment patterns by EOCG PS status.

**Figure 1** shows 1L treatment utilization by age group and ECOG performance status in the pre- and post-IO period. Notably, in the post-IO period, anti-PD-1/L1 monotherapy constituted the majority of systemic treatments utilized in the 1L setting for ≥75-year-olds (USON, 67%; IQVIA, 50%) and for those with ECOG≥2 (USON, 63%; IQVIA, 55%).

## 4 Discussion

To our knowledge, this is the first study to evaluate the temporal changes in mUC patient characteristics and 1L treatment receipt following the approval of anti-PD-1/L1 treatments. We demonstrate, over the last decade, an increasing proportion of mUC patients older than 75 years of age and with ECOG PS of 2 or higher among those treated in the 1L setting, reflecting an older and frailer population. Further, after approval of 1L anti-PD-1/L1 treatment for mUC, there is a decrease in platinum-based treatments and non-NCCN recommended therapies as 1L treatment, and a marked increase in the use of anti-PD-1/L1 monotherapy. Finally, anti-PD-1/L1 monotherapy constituted the majority of systemic treatment starts in the 1L setting for patients 75 years of age or older and for those with ECOG PS 2 or higher.

mUC predominantly affects the elderly. Older patients are much more likely to present with increased comorbidities and decreased renal function, which may affect treatment choices and willingness to accept treatment toxicity. In this population, data from two large U.S. cohorts suggest that the anti-PD-1/L1 monotherapies are therapies of choice and fulfill a critical unmet need among older and frailer mUC patients who are ineligible for platinum-based therapies. This study has several limitations. Data from USON and IQVIA EMR databases are mostly derived from community oncology practices and may not necessarily generalizable to the US population. A considerable group of patients had missing data on ECOG PS. Future studies with updated data are warranted to further understand the 1L changing landscape and outcomes for mUC patients.

**Take home message:**

This real-world evidence study suggests that recently approved immuno-oncology medications have filled a therapeutic gap in first-line advanced/metastatic urothelial cancer patients, especially for older and/or frail patients.

## Data availability statement

The datasets presented in this article are not readily available because the study included de-identified data and it was analyzed within the data owner systems. Requests to access the datasets should be directed to the corresponding author.

## Ethics statement

The studies involving humans were approved by McKesson Compliance/Privacy department and the Institutional Review Board (IRB). The studies were conducted in accordance with the local legislation and institutional requirements. Written informed consent for participation was not required from the participants or the participants’ legal guardians/next of kin in accordance with the national legislation and institutional requirements.

## Author contributions

GD, HL, and MB contributed to conception and design of the study. SA and KW performed the statistical analysis. HL and MB wrote the first draft of the manuscript. All authors contributed to the article and approved the submitted version.
