# Supplementary material for: Temporal changes in treatment patterns by age group and functional status before and after PD-1/L1 inhibitor approvals in advanced urothelial carcinoma
Source: Front Oncol. 2023 Oct 2;13:1210208. doi: 10.3389/fonc.2023.1210208 (PMC10577172; doi:10.3389/fonc.2023.1210208)
Supplement: Supplementary file 1 [file Table_1.docx]

Supplementary Material

**Temporal Changes in Treatment Patterns by Age Group and Functional Status Before and After PD-1/L1 Inhibitor Approvals in Advanced Urothelial Carcinoma**

**Gurjyot K. Doshi^1^, Haojie Li^2^, Mehmet Burcu^2^, Srinivas Annavarapu^3^, Karen Wells^4^, Kentaro Imai^2^, Blanca Homet Moreno^2^, Puneet Singhal^2^, Ronac Mamtani^5^**

*** Correspondence:** Haojie Li: [haojie.li@merck.com](mailto:haojie.li@merck.com)

**Table 1.** Baseline Characteristics: Real-world 1L Treated Advanced Urothelial Carcinoma Patients

|  | US Oncology (USON) EMR | | | IQVIA Oncology EMR | | |
| --- | --- | --- | --- | --- | --- | --- |
|  | (Jan 2010-Mar 2017) | (Apr 2017-Sep 2020) | (Jan 2012-Mar 2017) | | (Apr 2017-Sep 2020) | |
| Total patient count | 1508 | 928 | | 1130 | | 1391 |
| Age at 1L treatment initiation |  |  | |  | |  |
| Mean (SD) | 70.8 (10.5) | 71.6 (11.0) | | 69.3 (9.5) | | 71.6 (9.4) |
| Median | 71 | 72 | | 71 | | 73 |
| IQR | 63, 78 | 64, 80 | | 63, 77 | | 66, 79 |
| Min, Max | 26, 90+ | 28, 90+ | | 29, 84+ | | 28, 84+ |
| Age group |  |  | |  | |  |
| < 65 | 422 (28.0%) | 242 (26.1%) | | 324 (28.7%) | | 308 (22.1%) |
| 65 - 74 | 520 (34.5%) | 303 (32.7%) | | 388 (34.3%) | | 457 (32.9%) |
| 75+ | 566 (37.5%) | 383 (41.3%) | | 418 (37.0%) | | 626 (45.0%) |
| Gender |  |  | |  | |  |
| Female | 388 (25.7%) | 245 (26.4%) | | 292 (25.9%) | | 368 (26.6%) |
| Male | 1120 (74.3%) | 683 (73.6%) | | 837 (74.1%) | | 1013 (72.4%) |
| Unknown |  | 0 | | 1 | | 10 |
| Race |  |  | |  | |  |
| White | 1071 (92.5%) | 701 (89.0%) | | 689 (90.3%) | | 448 (86.3%) |
| Black Or African American | 55 (4.7%) | 66 (8.4%) | | 38 (5.0%) | | 29 (5.6%) |
| Other | 32 (2.8%) | 21 (2.7%) | | 36 (4.7%) | | 42 (8.1%) |
| Not documented | 350 | 140 | | 367 | | 872 |
| ECOG at 1L treatment initiation |  |  | |  | |  |
| 0-1 | 908 (80.9%) | 450 (75.4%) | | 307 (85.5%) | | 550 (79.3%) |
| 2+ | 215 (19.1%) | 147 (24.6%) | | 52 (14.5%) | | 144 (20.7%) |
| Not documented | 385 | 331 | | 771 | | 697 |
